# Integrity over fidelity: transformational lessons from youth participatory action research to nurture SEL with adolescents

**DOI:** 10.3389/fpsyg.2023.1059317

**Published:** 2023-07-21

**Authors:** Emily Anne Meland, Gretchen Brion-Meisels

**Affiliations:** Harvard University, Cambridge, MA, United States

**Keywords:** social emotional learning, youth participatory action research, critical participatory action research, fidelity of implementation, adolescent development

## Abstract

Much has been written about social and emotional learning (SEL) and its positive impact on young people’s academic and life outcomes, yet most of this research is based in early childhood and elementary settings. SEL programming for adolescents has shown mixed results, with many programs proving to be largely ineffective or even showing slightly negative impacts for some youth. Adherence to scripted SEL curricula, or “fidelity” to the program components, is often seen by young people to be “lame”, inauthentic, and condescending, failing to connect to the topics and issues that feel most critical to them in this stage of their development. For all students, and especially for those whose identities have been systematically marginalized or oppressed by the dominant culture, SEL programming that fails to explicitly address these experiences of injustice often feels inauthentic and out of touch for youth. Therefore, effective implementation of SEL for adolescents is likely to require skillful adaptation and responsiveness to the identities, interests, and motivations of students by educators. In this case, effective SEL may look less like fidelity to a specific set of scripts, sessions, or activities, but rather a commitment to the wholeness of a set of core principles, relationships, and opportunities for adolescent exploration and leadership/empowerment, or what we will call *integrity* of implementation. In this paper, we present one promising approach to adolescent social and emotional development – youth participatory action research (YPAR) – and the ways in which studying the YPAR process (in addition to the research topics selected by youth) can provide key insights into the social and emotional learning and development of youth.

## Introduction

In recent decades, there has been a growing consensus about the contributions that high quality social and emotional learning (SEL) can make to young people’s positive life outcomes (Durlak et al., 2011; Jones and Kahn, 2017). While SEL programming likely supports both children and adolescents, most of the research on effective SEL interventions is based on work done in early childhood and elementary settings (Domitrovich et al., 2017; Yeager, 2017). In these settings, social and emotional learning often looks like a decontextualized, or predetermined, set of lessons and activities that have previously demonstrated a positive impact on students’ social and emotional skills (Jones S. M. et al., 2017). This preset or “boxed” approach to social and emotional learning has shown mixed results with adolescents; in large-scale studies, many programs seem to be largely ineffective or even show slightly negative impacts for some youth (Ciocanel et al., 2017; Domitrovich et al., 2017; Yeager, 2017). Qualitative data suggests that adherence to scripted SEL curricula, or “fidelity” to the program components, is often seen by young people to be “lame” (Sawchuk, 2021), inauthentic, and condescending, failing to connect to the topics and issues that feel most critical to them in this stage of their development (Yeager, 2017). This is likely related to the unique developmental needs of adolescence – a growing need for autonomy, identity exploration and resolution, and relationships that provide a sense of belonging – which strict fidelity to an SEL program may undermine or fail to address (Roeser et al., 2000; Ciocanel et al., 2017; Domitrovich et al., 2017; Yeager, 2017; Jagers et al., 2019; Owens et al., 2022).

Faced with this set of challenges, understanding SEL implementation beyond *fidelity to a manual* or *set of scripts* – at the level of its essential mechanisms of change – becomes essential (O’Donnell, 2008; Abry et al., 2015; Jones S. M. et al., 2017). A 2011 meta-analysis of over 200 school-based SEL programs showed that implementation is a key moderator of evidence-based SEL program outcomes. With academic and social emotional impacts almost twice as large for programs that were implemented effectively, as compared to those that encountered problems with implementation (Durlak et al., 2011; Durlak, 2016). Yet, even in this meta-analysis, only 57% of the studies monitored implementation at all, and implementation problems encompassed any implementation issues reported by the authors. This points to the challenge of studying implementation, which itself is a multi-dimensional construct around which the field continues to theorize. We must more precisely understand what aspects of implementation are critical to impacting SEL program outcomes (e.g., Dusenbury et al., 2003; Durlak and DuPre, 2008; Berkel et al., 2011; Proctor et al., 2011; Durlak, 2016).

In this paper, we share some insights from youth participatory action research (YPAR), which we believe is an approach to research, youth development, and systems change that promotes social and emotional skill development by providing adolescents with tools, relationships, and collective opportunities to advocate for more just and equitable environments. As a result, YPAR not only impacts the environments in which youth live, but can also provide youth with feelings of autonomy, connection, and agency. While widely studied as an onto-epistemological approach to research, the lessons of youth participatory action research are often overlooked in the field of social and emotional development. There is limited research connecting these two fields of work or helping them to learn from each other. To some extent, this gap may be the result of epistemological differences in how YPAR and SEL researchers tend to design their studies and conceptualize their outcomes. YPAR studies tend to be critically oriented and focus on setting-level outcomes that indicate improvements in educational equity or justice, whereas many SEL studies seek to measure the efficacy of a program through the aggregated individual-level outcomes of students. Despite this, we believe that there are critical lessons that the field of SEL can learn from youth participatory action research.

In this article, we share one important lesson about how social and emotional learning might be better understood if we were to measure *integrity of implementation* over fidelity, drawing from LeMahieu (2011). LeMahieu (2011) cites the need for “less fidelity of implementation (do exactly what they say to do) [and] more integrity of implementation (do what matters most and works best while accommodating local needs and circumstances)” in implementation science. Identifying what matters most to nurturing social emotional development and wellbeing – the true active ingredients or “kernels of SEL” for youth (Jones and Bouffard, 2012; Li and Julian, 2012; Jones S. et al., 2017) – allows us to shift our conceptualization away from fidelity to standardized activities and toward culturally and contextually responsive *integrity*. This is something we can learn by studying the YPAR process, which grounds itself in a set of core onto-epistemological principles, or commitments.

## Moving from fidelity to integrity

One challenge in the implementation science literature is that we lack a clear set of operational definitions for the different aspects of implementation that we might hope to measure (Dusenbury et al., 2003; Durlak and DuPre, 2008; Proctor et al., 2011). For example, fidelity has been used interchangeably with terms such as “adherence,” “compliance,” “integrity,” and “faithful representation” (Durlak and DuPre, 2008, p. 329). These terms and how they are operationalized may be interpreted differently by both researchers and practitioners, ranging from perfect adherence to a scripted and sequenced set of activities, to implementation of a program to an acceptable level or target compliance rate, while allowing for some changes or adaptation (Durlak and DuPre, 2008; O’Donnell, 2008; Berkel et al., 2011). We argue that further clarity and distillation of the fidelity concept is necessary. Future conceptualizations must distinguish between fidelity as defined as “the degree to which an intervention was implemented as it was prescribed in the original protocol” (Proctor et al., 2011, p. 69), and integrity, defined as the degree to which an intervention was implemented maintaining its core active ingredients, while authentically and fully integrating the assets and needs of the local community. In our work researching SEL implementation with K-12 teachers, we find that practitioners grapple with this tension in tangible ways. Teachers have expressed worries to us that if they do not follow a set of program scripts word-for-word, they may undermine the efficacy of the research-based program being studied. Yet, in trying to stick so closely to the script and carry out the intervention “with fidelity,” these same educators may undercut the authenticity through which they execute the program and overlook opportunities to be responsive to the backgrounds, needs, and interests of their students. In doing so, they actually miss the critical active ingredients of the intervention (e.g., the development of authentic and reciprocal relationships). It is also common for teachers to share that SEL programs do not resonate with and are not responsive to their students. Without being privy to and having a clear understanding of the theoretically important program components, practitioners are not confident as to when they can and cannot deviate from the script, even to make changes that may make the program more effective for their own students. We hypothesize that increased integrity of implementation would positively impact implementation quality, defined as “the processes used to convey program material to participants “(Berkel et al., 2011, p. 26). Providing teachers with the tools to know when and how to adapt the curriculum to meet the needs and interests of their students and the strengths of their teaching practice, while maintaining integrity of implementation, has the potential to positively impact all components of quality of process, including: “(1) teacher–student interactivity, (2) teacher enthusiasm, (3) teachers’ communication of goals and objectives, (4) student engagement, (5) student attentiveness, and (6) students expressing their opinions” (Dusenbury et al., 2005, p. 310). Thus, specifying the theoretically important program components – often called active ingredients, or “kernels of SEL” – becomes critical (Durlak and DuPre, 2008; Berkel et al., 2011; Abry et al., 2015; Durlak, 2016; Jones S. et al., 2017).

This tension has often been framed in the implementation science literature as a tension between fidelity and adaptation, a debate which first challenged the relevance of strict fidelity of implementation to program success (Durlak and DuPre, 2008; Berkel et al., 2011; Durlak, 2016). Indeed, studies have shown that adaptations during implementation can improve the effectiveness of interventions, by potentially “increasing: ownership on the part of community implementers, perceived relevance on the part of participants, and the match between the program and the ecological niche” (Berkel et al., 2011, p. 26). Yet, not all adaptations are associated with program outcomes. Studies thus far have shown that additions to programs, in the context of high fidelity, are associated with improved outcomes, but changes or modifications in the absence of fidelity, are typically not (Berkel et al., 2011). More research is needed to understand “whether and under what conditions adaptation or reinvention might enhance program outcomes, and under what conditions adaptation or reinvention results in a loss of program effectiveness” (Dusenbury et al., 2003, p. 252). We believe that *integrity* of implementation in SEL, or the degree to which an intervention was implemented maintaining its core active ingredients, while authentically and fully integrating the assets and needs of the local community, would allow us to see more clearly inside the “black box” of implementation. It would allow us to understand why and how adaptations can contribute to program effectiveness, and preempt when they would fall short (Durlak, 2016). Indeed, adaptations to increase program efficacy become the expectation, rather than a deviation. With integrity of implementation, it is made clear to those delivering the intervention exactly what matters most for young people’s social and emotional development, those core components without which we would not expect to see change. This is also what should be described and measured in evaluations, increasing our understanding of the true mechanisms of change in our interventions. From there, while the program may provide suggested activities, adaptation to the local context is encouraged, expected, and supported through the program design and implementation, rather than seen as a deviation from the program’s intent.

The distinction between fidelity and integrity may help us to better understand the research on program adaptation that shows a positive correlation between adaptation and program efficacy (Durlak and DuPre, 2008). For example, decades of research points to the importance of relationships as the active ingredient, or key mechanism, in human development (Li and Julian, 2012; Osher et al., 2018). In studying program implementation, Durlak and DuPre (2008) describe findings from a Mitchell (1983) study of youth mentorship that “found that the types of activities performed during a mentoring program were unrelated to outcomes, perhaps because the quality of the relationship formed between mentor and youth was more important. In mentoring, it may not be what you do but how you do it that counts” (p. 341). In this case, integrity of implementation, specifically with regard to the development of authentic relationships between youth and their mentors, was the most critical lever of change for positive youth development in the mentorship program. When it comes to SEL with adolescents, integrity of implementation may trump fidelity of implementation in promoting positive youth development. While currently, “efforts to empirically validate hypothesized core components are quite rare” (Berkel et al., 2011, p. 25), we argue that this is an essential piece of effective SEL research and implementation.

## Social emotional learning

Social emotional learning (SEL) commonly refers to the process through which people acquire skills, attitudes, behaviors, and values essential for success in school, work, and life (Jones and Bouffard, 2012). These skills and competencies can primarily be grouped into three large buckets, or domains: cognitive, social, and emotional (Jones and Kahn, 2017). In addition, SEL is considered to include the development of mindsets, character, values, and identity (Jagers et al., 2019; National Commission on Social, Emotional, and Academic Development, 2019). Definitions and measures of social and emotional skills often vary across programs, sometimes making it challenging to isolate the effects of SEL programs on specific social and emotional skills and competencies (Jones and Doolittle, 2017; Jones et al., 2019). Despite this, there is an extensive body of literature linking SEL programming to individual-level outcomes, such as academic performance, behavior, mental health, and positive youth development (Durlak et al., 2011), and fewer studies linking SEL to teacher, classroom, school, or community-level outcomes (Jones S. M. et al., 2017).

Decades of research in developmental science tells us that social and emotional skills build and become increasingly complex over time, and that more basic social and emotional skills learned in early and middle childhood become the building blocks for more complex social emotional skills and competencies in adolescence and adulthood. For example, children must first learn to identify and name their own and others’ emotions before they are able to acquire more complex problem-solving and perspective-taking skills (Jones S. M. et al., 2017). This means that both the targeted skills and the ways in which they are taught must be aligned with a young person’s age and stage of development, and that SEL should support young people in meeting the unique demands of their contexts (Jones S. M. et al., 2017). What is often overlooked is the importance of culturally responsive and sustaining SEL (Jagers et al., 2019; Meland et al., 2019), which requires that educators align their curriculum with the cultural strengths and contextual experiences of youth in order to honor and sustain students’ diverse cultures and ways of being, while simultaneously disrupting systems of oppression that privilege certain ways of being (e.g., White, middle-class, heteronormative, and neurotypical, etc.) over others. For example, many SEL programs fail to explicitly address structural inequity, rendering themselves inauthentic for youth (Kaler-Jones, 2020). While calls have increased in recent years for SEL that is transformative, fearless, abolitionist, liberatory, and humanizing (Jagers et al., 2019; Simmons, 2021; Camangian and Cariaga, 2022; DeMartino et al., 2022), the systematic translation of these ideas into SEL classroom practice has not been actualized. This lack of cultural and contextual responsiveness often leaves adolescents with SEL programming that feels out of touch. Unfortunately, as Jones S. M. et al. (2017) point out, “SEL programs and interventions frequently target the same skills in the same ways across multiple years” (p. 52), exacerbating this issue. For adolescents, whose social and emotional skills and their applications are becoming increasingly integrated and complex, the approaches to SEL that were used in their early years no longer meet the developmental moment.

Effective implementation of SEL for adolescents requires skillful adaptation and responsiveness to the experiences, interests, and motivations of students, and must explicitly attend to the stage-salient tasks of fostering identity development, agency, and belonging (Jagers et al., 2019). This type of adaptation can be planned for with the creation of flexible curricula, but requires that educators and youth have control over the specific content of local activities. When conceptualizing high-quality implementation of SEL programming for adolescents in this way, it is especially important to understand conditions of the environment that support or hinder social and emotional development. In other words, each local context and moment brings its own challenges to health and wellness for young people, requiring a nuanced set of social emotional skills to navigate. Often, there are structural factors that create systemic inequity, which contribute to these challenges, as well as interpersonal and internal dynamics. Given this reality, effective SEL may look less like fidelity to a specific set of scripts, sessions, or activities, but rather a commitment to a set of core principles, relationships, and opportunities for adolescent exploration and empowerment. This is what we refer to as *integrity of implementation.*

## Youth participatory action research as a means of nurturing adolescent social and emotional development

One promising approach for fostering adolescent social and emotional development, which capitalizes on the opportunities and strengths of this developmental period *and* responds to cultural and contextual factors, is Youth Participatory Action Research (YPAR) (Cammarota and Fine, 2008; Mirra et al., 2016; Ozer, 2017; Fine and Torre, 2019; Jagers et al., 2019; Ozer et al., 2020). We do not believe that YPAR should be instrumentalized as another version of an SEL program, since it is intended as an approach to research and social change. However, understanding the YPAR process can provide key insights into the active ingredients or “kernels” of adolescent social and emotional learning that take place through this type of engagement. We might better understand integrity of implementation in SEL through looking at the YPAR process, because its implementation centers on a set of core commitments rather than a set of predefined activities to accomplish its aims.

YPAR is a form of participatory action research (PAR)^1^ in which youth are full participants in the research process and seen as the experts of their own lives and contexts (Caraballo et al., 2017). Youth identify topics of inquiry relevant to their life experiences, in which they may interrogate the structural, interpersonal, and psychological factors influencing their lives, collect data on these topics, and engage in systemic analysis. This work is supported by the presence of trusted adults who are knowledgeable of the research process, and who often facilitate or teach some specific research tools, and partner with youth through democratic participation in this process. Ultimately, YPAR projects seek to create some form of collective action that aims to disrupt systems of inequity and promote positive change in communities (Rodríguez and Brown, 2009; Mirra et al., 2016; Brion-Meisels and Alter, 2018). These action projects may take many forms including public art, presentations, recommendations to school and community leaders, and other forms of advocacy. YPAR harnesses the energy, passion, and potential of this critical developmental period by providing youth the chance to build strong relationships with adults and peers, better understand themselves and their communities, study sociopolitical questions of interest, and take action on issues that affect their lives (Rodríguez and Brown, 2009; Ozer, 2017). Through this process, youth have the opportunity to build and display a myriad of social emotional skills, including communication, collaborative problem-solving, critical thinking, identifying and managing emotions, empathy and perspective-taking, and civic values/participation (Ozer et al., 2020).

Despite sharing goals and outcomes around positive youth development, the fields of SEL and YPAR have not always been in dialogue, in part because of differences in their epistemological and ontological origins. Research on the impact of YPAR on SEL specifically has been limited, but studies of YPAR in school settings have shown that YPAR can promote the development of critical thinking skills (e.g., Kirshner et al., 2011), support sociopolitical development (e.g., Cammarota and Romero, 2011; Zeal and Terry, 2013), increase the diversity and depth of social connections (e.g., Flores, 2007), and increase youth voice in school-based decision making (Mitra, 2008; Kirshner et al., 2011; Chou et al., 2015). Studies of YPAR often utilize mixed-methods approaches to understand the impacts of YPAR on structural and cultural aspects of a setting, and sometimes also study its effects on youth over time. For example, in the largest quasi-experimental study of school-based YPAR, researchers found that adolescents who were randomly assigned to the YPAR class “showed increases in sociopolitical skills, motivation to influence their schools and communities, and participatory behavior” as compared to the control group who took part in a direct service peer mentor project (Ozer and Douglas, 2013, p. 66). Future studies of the YPAR *process* (distinct from the research that the youth and their co-researchers conduct in their communities) may consider measuring changes in youth social and emotional competence to further our understanding of these links.

YPAR projects each engage a unique set of research questions and processes that are responsive to the context and priorities that youth participants identify. Unlike in traditional SEL research, where outcomes are often predetermined based on research-driven priorities, in these projects, the outcomes under investigation are generated by the youth/community, and the set of research activities undertaken depends on the research questions. Therefore, fidelity to a specific set of activities cannot be fixed, or pre-determined; rather, the YPAR process must be fluid and adaptive to meet the questions and needs of the communities in which the projects are carried out. In this way, YPAR projects vary significantly in their content and chosen outcomes. At the same time, most of these projects share a set of core commitments that guide the YPAR process. These commitments, outlined below, might be seen as “active ingredients” or “kernels” of the work (Li and Julian, 2012; Jones S. et al., 2017) – they help us to identify *why* YPAR projects often nurture adolescents’ SEL skills and to understand *how* these projects nurture SEL in culturally and contextually responsive ways. In the remainder of this paper, we outline these core commitments and propose that studying *integrity* of implementation may provide us with important information about the mechanisms through which youth build social and emotional skills in the YPAR process.

## A focus on core commitments

Scholars in the field of participatory action research (PAR) have described the core principles and commitments of the work in different ways (see Rodríguez and Brown, 2009; Torre et al., 2012; Brion-Meisels and Alter, 2018; Cammarota et al., 2018). In this paper, we choose to summarize the commitments identified by scholars in three broad buckets, or groups. By describing the commitments in this way, we intend to help SEL scholars draw explicit connections between the commitments of Y/C/PAR and the mechanisms of transformation for youth (see Table 1). In addition to these three buckets/groups of commitments, we add a fourth bucket focused on the commitment to authentic and reciprocal relationships.^2^ From this point forward, we will refer to these as the YPAR commitments to highlight our focus on youth development, while recognizing that these commitments are grounded in critically-oriented approaches to participatory action research.

**Table 1:** Mechanisms of transformation through YPAR commitments.

YPAR commitment	Mechanism of transformation^*^	Assessing integrity
**Content.** The YPAR team agrees to the interrogation of real-life, relevant issues identified by youth, at least in part through the lens of the structural factors that promote or inhibit community thriving.	Engagement in content that is culturally and contextually relevant (Ladson-Billings, 1995; Paris, 2012) Critical consciousness development (Freire, 1973; Seider and Graves, 2020) Motivational processes– competence, autonomy, relatedness (Roeser et al., 2000; Eccles and Wigfield, 2002)	Review of research questions for relevance to students’ lives and opportunities to interrogate power and/or structural inequity Observing the process through which students arrive at their topic of inquiry Engaging youth in conversation (e.g., focus groups, interviews, photovoice, video reflection)
**Process.** The YPAR team agrees to deep and democratic participation in which youth expertise is essential and those most impacted by the research are centered in its design.	Reciprocal engagement (Li and Julian, 2012; Osher et al., 2018) Shifting the balance of power toward young people (Sameroff, 2010; Li and Julian, 2012; Osher et al., 2018) Motivational processes – competence, autonomy, relatedness (Roeser et al., 2000; Eccles and Wigfield, 2002) Experiences of Competence and Confidence (Lerner and Lerner, 2013)	Reviewing documentation of participatory processes Observing the YPAR team Engaging youth in conversation (e.g., focus groups, interviews, photovoice, video reflection)
**Purpose.** The YPAR team agrees to engaging in collective action toward a more socially just community/world.	Deeper learning (Mehta and Fine, 2019) Critical consciousness development (Freire, 1973; Seider and Graves, 2020) Opportunities to display Character, Caring, and Contribution (Lerner and Lerner, 2013) Motivational processes – competence, autonomy, relatedness (Roeser et al., 2000; Eccles and Wigfield, 2002)	Reviewing documentation/ materials created for action Observing the YPAR team carry out their action projects Engaging youth in conversation (e.g., focus groups, interviews, photovoice, video reflection)
**Core.** The YPAR team agrees to authentic and trusted relationships amongst co-researchers, especially between youth and adult partners.	Caring, authentic, and reciprocal relationships (Valenzuela, 1999; Li and Julian, 2012; Osher et al., 2018)Sense of Connection (Lerner and Lerner, 2013)	Relationship surveys and self-reports Observing the YPAR teamEngaging youth in conversation (e.g., focus groups, interviews, photovoice, video reflection)

^*^Transformation takes place at both the setting and individual-level.

The first three commitments outlined below are grounded in the work of María Torre and her colleagues at the Public Science Project, who describe three epistemological commitments of critical participatory action research, each of which move the knowledge produced “toward a stronger validity” (Torre et al., 2012, p. 179) for those closest to the issue at hand. These commitments are: (1) “reframing the problem through critical theory” (ecological and construct validity), (2) “deep and broad participation” (expert validity), and (3) “action and accountability to social change movements” (impact validity) (p. 180). Grounding these commitments is a set of ontological beliefs, or assumptions, which underpin the intergenerational work. These include: “all people have valuable knowledge about their lives and experience; all people have the ability to develop strong critical analyses; all people have multiple identities and carry important histories, connections, and responsibilities to various communities; all people and institutions are embedded in complex social, cultural, and political systems historically defined by power and privilege; the production of knowledge is not objective, or value-free; social research is most valid using multiple/triangulated methods to help capture interconnected individual, social, institutional and cultural layers; participation is not automatic; and change is an ongoing process” (Torre, 2009). Rather than include each of these commitments separately in our table, we bucket them into groups that help illuminate how they nurture social and emotional development. In everyday practice, SEL is fostered throughout the YPAR process in complex and overlapping ways at both the setting and individual level. Our table over-simplifies this, for the purpose of helping scholars in the SEL field better understand the ways in which YPAR supports social emotional development for adolescents *and* how we might begin to assess integrity of implementation for each commitment.

Understanding the connection between each of the core commitments of YPAR and the central goals of SEL can help us to imagine a framework through which we might understand implementation in more iterative and flexible ways. In other words, if these commitments themselves are active ingredients of YPAR that nurture social and emotional development, then we can measure integrity with respect to these commitments, rather than fidelity to a specific set of activities.

Before sharing our thoughts about the ways in which SEL scholars might learn from the practice of YPAR, we believe it is important to share a bit about our own identities and backgrounds. The current perspectives emerged from our personal journeys as scholars committed to social and emotional development, adolescent agency/voice, and critically-oriented research. Each of us has spent time in K-12 settings as a classroom teacher, and each of us entered the world of academia because we believed that the tools of this world would help us better advocate for educational justice. Over time, we each became increasingly concerned with the ways in which traditional SEL research and practice placed dominant ways of being at the center of “good” social and emotional development – a critique that has been echoed by many others in the field (e.g., Jagers et al., 2019; Simmons, 2021; Camangian and Cariaga, 2022; DeMartino et al., 2022). This concern about SEL research is likely heightened by our positionalities, which provide us with significant social privilege in many contexts. [Author 1] identifies as a White, cisgender, heterosexual female with Italian immigrant ancestry who embodies many dominant social identities. She has benefited from contemporary educational systems that operate through the perpetuation of White supremacy, whether that be through what knowledge is valued, how success is measured, or what is deemed as an appropriate way to be and express in educational settings. As a classroom teacher, she was confronted most directly with the ways in which our U.S. school systems are often not set up to value and support students’ diverse backgrounds, experiences, and ways of being, setting her on a journey to learn and unlearn how to create educational spaces in which all children, youth, and adults can thrive. [Author 2] identifies as a queer White, cisgender female whose ancestors were a part of the Jewish diaspora, and who has benefited economically from contemporary educational systems. She, also, is working to unlearn colonial ways of being, which is a challenging process that pushes her to slow down, decenter her own thinking, and recenter embodied ways of knowing.

In the following sections we explore each commitment within the context of a YPAR project, and then define how it might help us think about measuring integrity in SEL with adolescents.

## Commitment one: youth driven and contextually relevant content

The first commitment describes the *content* of the project. The focus of inquiry in YPAR projects is on issues identified by youth as impacting their lives, viewed through the lens of critical theory (Torre et al., 2012). Rodríguez and Brown (2009) describe this as “a commitment to research and learning in which the topics of inquiry, the content of learning, and the knowledge produced reflect and address the real life problems, needs, desires, and experiences of youth researchers” (p. 24–25). These issues should be ones that can be interrogated through the lens of the structural factors that promote or inhibit community thriving. As Torre et al. (2012) explain, “Critical inquiry deliberately shifts the gaze from ‘what’s wrong with that person?’ to ‘what are the policies, institutions, and social arrangements that help to form and deform, enrich and limit, human development?’ and ‘how do people resist the weight of injustice in their lives?’” (p. 179).

In our work with youth, upholding this commitment begins with inviting young people to identify issues or opportunities that impact themselves and their communities, which they want to investigate. As adult partners, we serve as facilitators for this conversation, trying to ensure that all voices are heard, and we ask probing questions that help students interrogate the root causes of some of issues they identify. Because YPAR projects aim to investigate the structural factors that contribute to a given issue (in addition to the psychological and interpersonal factors), we often begin our brainstorming process with the visual metaphor of a tree. Youth are asked to brainstorm the ways they see, feel, and hear inequality in schools (the “leaves”) and things they think might be underlying or causing the inequality that they see (“the roots”). We then ask them to do the same exercise, but on a tree of liberation. What are examples of assets, strengths, moments of joy, or resistance to inequality that they have experienced in their educational journey (the “leaves”)? And what are the deeper structural, cultural, or institutional policies or practices that have supported these moments? Looking at all that they have brainstormed, students then consider what issues or opportunities they are interested in studying more deeply. Youth researchers might choose a topic for their research that aims to better understand one of the “leaves” and how it is connected to the roots in service of making their school a more equitable and liberatory space; or, they might choose to focus on a root, such that multiple leaves might be impacted.

Here is an example of this work in practice. One group of students at a working-class suburban high school noticed that their classes tended to be segregated, with wealthier and White students concentrated in the advanced placement classes, and less wealthy students and students of color in the regular tracks. In discussion, they called out similar trends in access across a range of school-based opportunities, as well as knowledge of and access to school-based supports (e.g., tutoring, mental health, guidance counseling). These students wanted to better understand which students had knowledge of how to access these opportunities and supports and/or found the supports useful, and why, so that they might propose ways to increase equitable access. This became the focus of inquiry for their project, upholding commitment one.

The tree activity is just one way to get students thinking about the issues they might want to address through YPAR; it is not the only way to honor this commitment. Reading across the literature on YPAR, one can find examples of projects where students have begun by studying social theory, and then extrapolated from the theory to consider their own context. One can find examples of projects where students have begun by talking about what frustrates or upsets them about their local context, and then dug into social theory about those issues. And, one can find examples of projects where a critical incident has propelled youth to action. What is important is that ultimately, the group selects a topic of inquiry that feels meaningful and relevant to the youth researchers and allows them to identify possible avenues to create change. By honoring this commitment, adult partners honor adolescents’ desires for autonomy and agency, as well as their naturally salient critical thinking skills (Roeser et al., 2000; Eccles and Wigfield, 2002). Regardless of *how* the students come to choose their topic, the process itself can scaffold mutual understanding and connection among participants by highlighting shared experiences and fostering empathy. It fosters perspective taking by giving young people (and adults!) an opportunity to listen to and learn from each other’s experiences. And, it can provide students with critical analytic tools that foster feelings of agency, as the team comes to consensus on what they want to study (or influence) in that context (Jagers et al., 2019).

Measuring integrity with regard to this commitment, rather than fidelity to the specific activities that it might entail, allows researchers to ensure that specific YPAR projects are including the mechanism necessary for social emotional learning to occur, while *also* providing local educators and organizers with the flexibility to design activities that best meet the cultural and contextual needs of their students. We believe that upholding this commitment increases the possibility that students will engage with topics that are culturally and contextually relevant (Ladson-Billings, 1995; Paris, 2012), harness their motivational processes (Eccles and Wigfield, 2002), and support the development of critical consciousness (Freire, 1973; Seider and Graves, 2020). At the setting level, this may represent a shift toward more youth-driven and culturally sustaining pedagogical practice (Paris, 2012; Ladson-Billings, 2014). Measuring this type of integrity of implementation could look like an analysis of the conversations that led to the project topic/question, an audit of the activities that students completed in order to pick their topic/question, or an analysis of how the research questions reflect the lived experiences of youth and their communities. It might also look like interviewing or surveying students after this step of the process, to understand how they viewed the adult partnerships to be upholding this first commitment, and how that commitment to youth- and community-driven content may have impacted their own thinking/behavior/sense of belonging. Ultimately, measuring the integrity of implementation for this commitment requires asking youth, as their perception of and connection to the research question may impact their motivation to engage in the YPAR activities and their sense of agency in creating positive change in their communities.

## Commitment two: participatory processes

The second commitment describes the *process* of YPAR projects, which requires deep and democratic participation from multiple stakeholders, with explicit efforts to amplify and center the voices of those most impacted by the issue under investigation (Torre et al., 2012; Brion-Meisels et al., 2020). This is described by Rodríguez and Brown (2009) as, “a commitment to genuinely collaborative methodological and pedagogical processes that validate, incorporate, and build on the knowledge and skills of youth researchers and support critical and creative engagement in research and learning” (p. 27). In this sense, knowledge is co-constructed in such a way that youth expertise is valued and viewed as essential to the validity of the process, and power is shared between youth and adults through democratic decision-making processes. Torre et al. (2012) further articulate that this process requires “co-constructing what questions most need asking; collaborating to develop both theory and method; [and] co-analyzing data” (p. 175).

In our work with youth, honoring this commitment has begun with collaborative decision-making about the issue/opportunity that they would like to investigate. In this early moment, we see our role as adult facilitators, in part, to ensure that all voices are given space and time. Often, we use this moment to talk about the ways in which research has harmed and helped communities in the past. As we teach students about what makes a good research question, and how the language in our research questions will guide our methodology, we unearth additional opportunities for participants’ voices to shape our process.

After collecting everyone’s ideas about the topic of inquiry, we work with students to refine the language of their research question. Using a protocol to gain consensus, we have students indicate “fist to five,” (National Center for Family Philanthropy, n.d.) whether they feel comfortable moving forward with the final language of the research question, or if we need to pause and continue to revise it. We have found that collaborative tools like Google documents, slides, and forms/surveys allow students to contribute their ideas and suggestions both in real time and asynchronously, after some time for reflection. Through this process, students are thinking deeply and critically about what they wish to understand, for whom, and how they will go about collecting data. As a team, we work to ask ourselves difficult questions about participation and voice – and wonder (aloud) about whose voices might be missing. After coming to consensus on a research question, we work with students to align their data-collection methodology to the question. Here, again, is an opportunity for students’ perspectives and prior knowledge to inform the shape of our project. For example, the students who wished to better understand how their peers gained access to opportunities and supports at their high school chose to create a school-wide survey to gather this information because they believed that this method would allow them to represent the most diverse range of voices. In the process of co-designing a study, adult partners and youth researchers have a chance to discuss many of the social and emotional dynamics in their local context, what participation means, and how different people can best access participation. These conversations often raise awareness about structural and interpersonal factors that shape wellbeing, as well as providing students with analytic tools to better understand their local context.

Once a study has been designed, the protocols themselves must be created. In the case of the project described above, this meant co-constructing a survey with youth researchers. Protocol creation and piloting is a time-consuming and arduous process, and different contexts require different levels of scaffolding and support. In our case, time constraints meant that we sometimes put sample questions in front of youth researchers to react to and to revise using language that would be most clear and accessible to their peers; while at other times, we invite students to develop their own survey questions. Every piece of the survey was reviewed and approved by the students through multiple rounds of review and discussion. Students then designed the recruitment strategy and set out to collect their data by encouraging their peers to take the survey and spreading the word through multiple channels (e.g., lunchroom tabling, email, Google classroom, school assemblies, etc.). Once their data had been collected, the students self-organized into groups to analyze various portions of the data. These groups discussed and came to a collective understanding of the key themes and interpretations of their findings. Finally, the students worked together to decide who would present what piece of information in their final presentation to the community. In each step of the analytic process, adult partners worked to provide students with the tools that they might need and to scaffold their ability to learn these tools; but the commitment to participatory processes required that the youth researchers collaboratively controlled the study design, analytic process, and findings. In this sense, the role of the adult partners was largely to continue to raise up questions about democratic participation and decision-making, provide students with models for how they might honor these commitments, and allow students to experiment with building a process that worked for their context.

Since SEL skills related to communication, collaborative problem-solving, decision-making, and planning (Jagers et al., 2019) are critical to this second YPAR commitment, measuring integrity of implementation for this commitment provides a flexible way of measuring whether the critical components that lead to setting-level and individual change/transformation are present. This commitment is supported in the implementation science and community psychology literature as well, which finds that “shared decision-making (community participation, collaboration) enhances implementation” and increases the chances that the program will be sustained over time (Durlak and DuPre, 2008, p. 340). We can imagine that participatory processes could be measured in multiple ways. It is possible to document instances of shared decision-making throughout the process, through observation or participant self-reporting. One might also interview youth or hold focus groups about their experiences with collaboration and collaborative decision-making. In addition to having these decision-making processes in place, we believe that it is equally important that youth feel that the process was truly democratic, and that they feel a sense of agency throughout the process. This can be documented through youth surveys or focus groups and triangulated with data on documented decision-making processes.

## Commitment three: purpose through collective action

The third commitment describes one of the central purposes of any YPAR project – to engage in collective action toward more socially just communities and societies. YPAR projects are designed to inform action to improve the lives of marginalized youth and their communities (Rodríguez and Brown, 2009). These actions might look like developing theory, engaging in social policy, “performing data” through arts-based methods, or making evidence available to organizing allies and activists (Torre et al., 2012). Research is conducted to understand and thereby take action against unjust systems that constrain the ability of all youth to thrive and to build up structures and supports that are protective and promotive.

In our work with youth, collective action has taken many forms. Often, youth research teams present their findings alongside actionable recommendations to those in positions of decision-making power in their communities – school leadership, policy makers, parents, and others. Sometimes, this first action leads to other actions, as the adults work with youth researchers to implement some of the recommendations proposed. Other times, collective action has taken the form of public art, photovoice projects, and community awareness campaigns – students have held events for their peers, designed infographics for school leaders, or provided professional development workshops to their teachers. Regardless of what collective action is taken, through the process of collective action, youth must think through the implications of their findings for different sub-groups. They must begin to develop theories about what lies underneath the findings – how routines, policies, structures, and interactions may be shaping different peoples’ experiences. And, they must cooperate to imagine how taking action might contribute to positive change.

Collective action requires that adults and youth practice a number of SEL skills, including demonstrating civic awareness and values, perspective taking, communication and consensus-building, planning and organizing, and adapting to shifts or changes in the plan. In the positive youth development literature, this might be framed as youth opportunities to demonstrate *character* by taking action to promote equity and social justice and *caring* for their communities, thereby leading to opportunities for *contribution* (Lerner and Lerner, 2013). Youth are supported to do so through the intergenerational research process in which adults can co-construct and scaffold skill-building.

While collective action is rarely considered explicitly in the implementation science literature, it builds from findings that empowering communities is an effective way to address local challenges, and that participation enhances implementation (Israel et al., 1998; Durlak and DuPre, 2008; Berkel et al., 2011; Proctor et al., 2011). Measurement of integrity for this commitment may look like documenting the action youth choose to pursue, and the process through which they agree upon this action. It might look like observing the conversations that youth researchers and adults have, as they come to consensus about the action steps they choose to take; or, asking youth to reflect on how the process of collective action shifted their ability to act in other settings (if at all). It is also important to assess, from the youth perspective, whether they believe that their actions can make a difference in their community, even in small ways, and that they are not simply going through the motions. This is important for the development of their civic consciousness (Sherrod et al., 2010), as well as their own wellbeing. Ultimately, much of the students’ experience in and perception of the YPAR project rests on the strength of the relationships that are formed throughout the process.

## Commitment four: relationships at the core

The fourth and final commitment describes the interpersonal conditions that drive learning throughout the YPAR process. We believe that, if the commitments above are embodied throughout the research process, then strong, trusting, and authentic relationships will form between co-researchers, especially between youth and adults. At the same time, we recognize that relationships are central to each of the commitments listed above, and forming these relationships builds over time and space. Relationships in which there is an ethic of care, reciprocity, scaffolded joint activity, and intentionality toward shifting the balance of power towards youth, provide the foundation for successful YPAR projects. Importantly, these relationships also support the positive social and emotional development of youth (Li and Julian, 2012; Osher et al., 2018). Given the essential role relationships play in positive youth development, we believe it is important to call out a focus on these relationships as the crucial fourth commitment, and a force that drives the other three.

In our work with youth, we prioritize commitment four by creating space for relationship building in each interaction with youth researchers. This might look like a fun icebreaker at the start of a meeting, checking in on what went well and what’s been challenging in our weeks, or demonstrating care around aspects of students’ lives outside of our project. This is common practice in SEL programming as well, grounded in a vast knowledge base on relationships as a core mechanism of youth social and emotional development (Li and Julian, 2012; Osher et al., 2018). Often, early in a project, we use games to practice collective decision-making, problem-solving, and action; debriefing these games can help scaffold our relationships and communication for future events. As a project moves forward, we are more likely to use check-ins to give students a chance to describe what they need from the community, how they are doing, and what is “up” for them on a particular day. We express interest and provide support for the other priorities in the young people’s lives as much as possible, sometimes forgoing the YPAR meeting agenda altogether so that the students might study for upcoming exams or prioritize other pressing commitments. As adults, we participate fully and model vulnerability in these activities; we consider ourselves co-researchers and team members. We work to ask for support when we need it, while carefully balancing our desire to be vulnerable with our desire to center young peoples’ voices and needs in the space.

Relationships are not only a critical mechanism of social and emotional development, but interpersonal relationships are also a crucial contextual factor that can enable or inhibit implementation (Lacouture et al., 2015). There is a vast body of literature on how researchers can measure quality relationships (Sabol and Pianta, 2012), but here, measuring integrity to this commitment means ensuring that this active ingredient/driver of transformation is present in YPAR projects, regardless of their specific content, methods, or collective action. Assessing integrity of implementation might look like administering a survey to students and adults regarding the developmental relationships they experienced through the project (e.g., Search Institute Developmental Relationships survey). It might also look like focus groups with students in which they are asked to reflect on the relationships built over the course of the project and how they believe they have impacted their trajectory. Additionally, it might involve asking students how their relationships on the research team have impacted their relationships to others outside the team (if at all).

## Contextual factors influencing integrity of implementation

As a final note, in documenting integrity of implementation in a YPAR project, it may be equally important to document the conditions of the environment (structural, interpersonal, political) that promote or constrain the ability for youth to carry out their projects and uphold the commitments described above. For example, youth researchers may encounter political resistance to their proposed collective action at the school or community level, or a lack of time or physical space may make it difficult to engage in fully participatory processes throughout the research project. The paper’s second author and others have written about the risks inherent in the “schoolification” of YPAR, which would need to be taken into consideration, should this approach be considered in a school-based setting (Brion-Meisels and Alter, 2018). These tensions include,

…authenticity around power sharing (Kohfeldt et al., 2011; Rubin et al., 2017); limited time, student, and staff turnover; imbalances of power (Rubin et al., 2017); centralized control over policies affecting the school (Kirshner, 2007; Ozer et al., 2008; Kohfeldt et al., 2011; Ozer and Douglas, 2013; Rubin et al., 2017); and student agency versus the structural constraints of schooling (Ozer and Douglas, 2013; Herr, 2017; Rubin et al., 2017). (Brion-Meisels and Alter, 2018, p. 432).

In this sense, partnering with youth in authentic research processes within the context of dominant institutional structures can be particularly challenging, and requires careful attention to upholding the core commitments of the process in the face of these tensions and constraints. Learning and unlearning will need to take place to support school-based adults to partner with, rather than act on or for, young people to understand, to understand the YPAR epistemology and what it means to systematically co-construct knowledge with youth, to address underlying adultism, to understand their positionality, power, and core purpose in carrying out this work with youth, and to embody the core commitments in order to conduct YPAR with integrity.

Understanding structural barriers to integrity of implementation provides critical information about the processes underlying the SEL outcomes we observe (Durlak, 2016). This is echoed by Lacouture et al. (2015), who describe:

…four layers of contextual factors that shape the implementation of the social programs: (1) the individual capabilities of the key actors to take the intervention forward (e.g., values, roles, knowledge, purpose), (2) the interpersonal relationships supporting the intervention (e.g., communication, collaboration, network, influences), (3) the institutional settings (e.g., informal rules, organizational culture, leadership, resource allocation, local priorities), and (4) the infra-structural system (e.g., political support) (p. 6).

In understanding integrity of implementation, we must also understand and document these critical contextual factors. All four of the factors described by Lacouture et al. (2015) above are relevant to the success of YPAR. With regard to the individual capabilities of key actors, YPAR is an approach to research built on the assumption that all human beings have the capacity and wisdom to engage in investigations of their lives. For this reason, academic “experts” or university researchers are not a necessary part of the YPAR process. Indeed, there are many projects that could be considered YPAR but tend to fall under the umbrella of community organizing because their primary purpose is action rather systematic study for generalizable knowledge as research is traditionally defined in academia. With that said, YPAR is a complex approach to the co-creation of knowledge that requires specific understandings about power, participation, and purpose, and therefore requires training, experience, and apprenticeship like any other approach to research or skill-development. This echoes the implementation science literature, which indicates at effective professional development is necessary for quality implementation, including an understanding of the theory behind an intervention and its core components or active ingredients (Durlak, 2016). As we have discussed in detail throughout this paper, the interpersonal relationships supporting YPAR are critical and central to the process. This is true not only for the adult and youth co-researchers, but of the relationships surrounding them, which may serve to support or to hinder or undermine the process. YPAR aims to explicitly impact the institutional and infra-structural contexts in service of creating more equitable communities; it is therefore influenced by and acts upon these features of the context, likely even more directly than the majority of traditional school-based interventions. Many of the same contextual factors that have been found to influence implementation quality are relevant considerations for the integrity of YPAR implementation; for further discussion of these factors from an ecological perspective see Domitrovich et al. (2008) and Durlak (2016). Future research may help us to better understand these conditions by explicitly studying questions of YPAR in a school-based context, including: What structural conditions need to change to enable adult-youth relationships in schools to flourish? What adult expertise and support is required to enact core commitments with integrity? And how might educators build relationships with students as co-conspirators in their search for justice when the educators themselves might also be the subject of student change efforts?

## Integrity over fidelity for authenticity and impact

In this paper, we share a set of commitments from the field of youth participatory action research (YPAR), which we believe contribute to the social and emotional development of youth in culturally, contextually, and developmentally aligned ways through both setting-level and individual transformation. It is important to note that YPAR was not designed as a social emotional learning intervention targeting individuals; quite the opposite, YPAR intends to create change at the organizational, institutional, cultural, or community-level, by supporting youth to investigate and act upon the forces that shape their lives. Still, through integrity of implementation in the YPAR process – upholding a set of core commitments – we see that both setting-level transformation and individual-level social emotional learning often take place. This is likely because measuring integrity of implementation gets us much closer to understanding the key active ingredients and mechanisms of change at both of these levels. We believe that this is an important lesson for the field of social emotional learning. Perhaps it is not by understanding fidelity to a set of predetermined activities, but rather integrity to a set of core principles or commitments, that we can glean more powerful insights into the drivers of social emotional learning and development for adolescents.

## Author contributions

EM and GB-M contributed conception and design of the paper. EM wrote the first draft of the manuscript. GB-M reviewed and wrote sections of the manuscript. All authors contributed to the article and approved the submitted version.

## Conflict of interest

The authors declare that the research was conducted in the absence of any commercial or financial relationships that could be construed as a potential conflict of interest.

## Publisher’s note

All claims expressed in this article are solely those of the authors and do not necessarily represent those of their affiliated organizations, or those of the publisher, the editors and the reviewers. Any product that may be evaluated in this article, or claim that may be made by its manufacturer, is not guaranteed or endorsed by the publisher.

## Footnotes

^1^YPAR, PAR, and CPAR can all be used to describe an approach to participatory action research (PAR). In this paper, we use YPAR to refer to intergenerational critical participatory action research with youth, highlighting the key role adolescents play in the process, and how this collaboration with youth might impact their social and emotional development. We also reference the CPAR commitments, as we believe that a core principle of YPAR is its critical engagement with issues of power and social justice.

^2^A commitment to trusting, equitable, and reciprocal relationships underlies all high quality Y/C/PAR work and is threaded throughout the process. We pull out this commitment as a separate bucket, because of its centrality in SEL.
